# Supplementary figures and images for: Description and spatial inference of soil drainage using matrix soil colours in the Lower Hunter Valley, New South Wales, Australia
Source: PeerJ. 2018 Apr 16;6:e4659. doi: 10.7717/peerj.4659 (PMC5907776; doi:10.7717/peerj.4659)

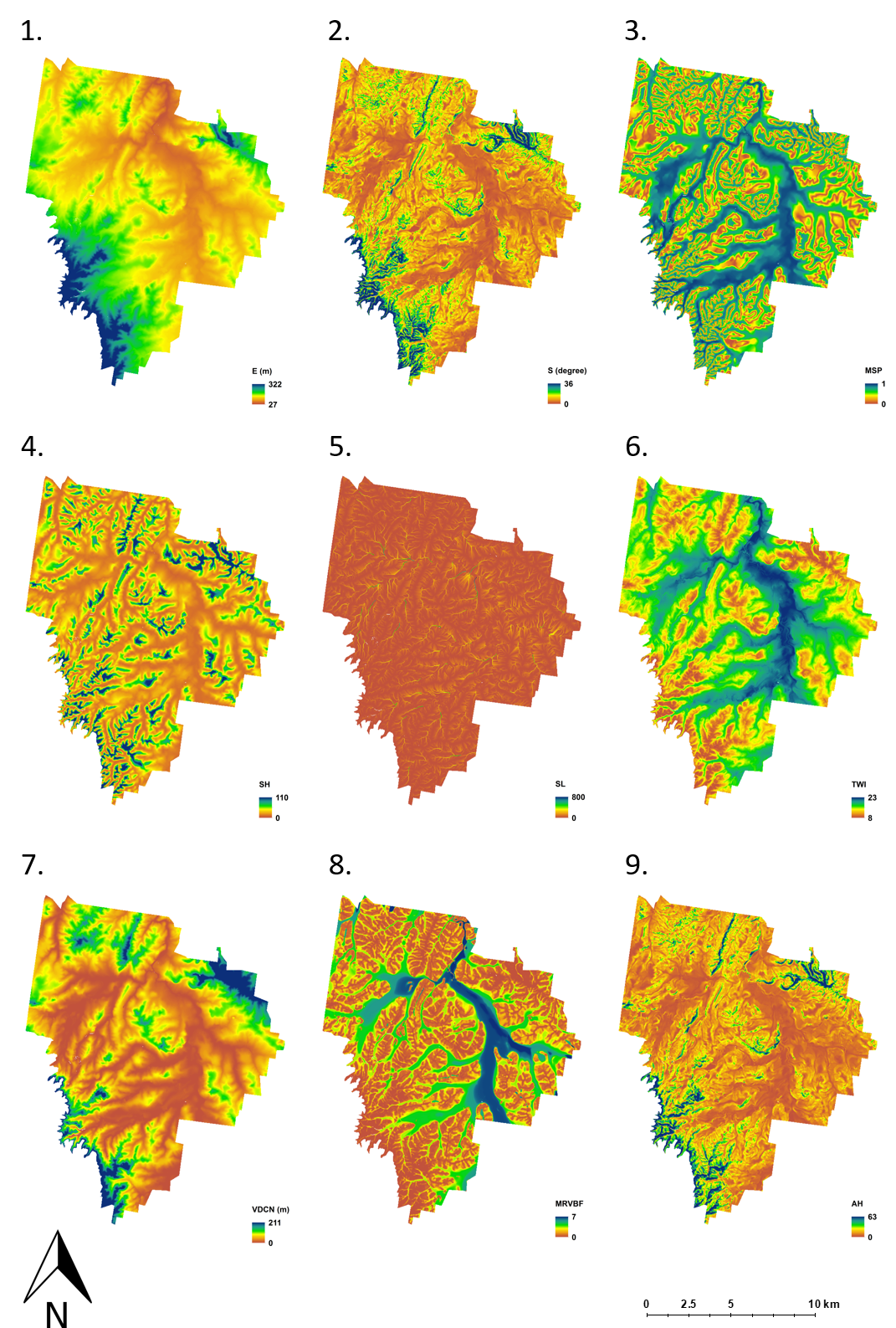

Supplement: Supplemental Information 1 — Topographic variables used in this study. 1. Elevation (E), 2. Slope gradient (S), 3. Mid-slope position (MSP), 4. Slope height (SH), 5. Slope length (SL), 6. Terrain wetness index (TWI), 7. Vertical distance to channel network (VDCN), 8. Multi-resolution valley bottom flatness (MRVBF), 9. Analytical hillshading (AH). [file peerj-06-4659-s001.png]
